# Glomangiopericytoma: a case series with review of literature^☆^

**DOI:** 10.1016/j.bjorl.2021.02.007

**Published:** 2021-03-06

**Authors:** Aasif A. Kazi, Elizabeth M. McDougal, Jessica B. Howell, Theodore A. Schuman, Ryan S. Nord

**Affiliations:** a Virginia Commonwealth University, Department of Otolaryngology – Head and Neck Surgery, Richmond, United States; b University of South Carolina, School of Medicine, Columbia, United States; c Otolaryngology Associates of Tennessee, Nashville, United States

## Introduction

A rare sinonasal tumor, glomangiopericytoma (GPC) was first described in 1942 by Stout and Murray under the now more encompassing term hemangiopericytoma.^1^ Similar to all hemangiopericytomas, GPCs originate from pericytes, however, the latter distinctly arises in the sinonasal region and from the pericytes of Zimmerman. These low-malignant-potential neoplasms are localized to the nasal cavity, with paranasal involvement and skull base extension uncommon.^2^ If paranasal sinus involvement is present, the sphenoid and ethmoid sinuses are most commonly affected.^3^ Symptoms are due to mass effect and include headache, nasal congestion, epistaxis, and sinusitis.^2^ There is no clear etiology for GPC, but they have been associated with pregnancy, hypertension, previous trauma, and use of corticosteroids.^3^ Additionally, Lasota et al. demonstrated that these neoplasms have overexpression of B-catenin and cyclin D1, supporting their role in the pathogenesis of GPC as well as the formers utility as a diagnostic marker.^4^, ^5^ These tumors account for less than 0.5% of all sinonasal neoplasms and generally have an excellent prognosis after complete surgical excision.^6^ We describe a series of cases of GPC presenting as nasal obstruction, hyposmia, and recurrent epistaxis.

## Case series

### Case 1

A 51-year-old male with a history of coronary artery disease, hypertension, chronic sinusitis, and tobacco abuse presented with complaints of right-sided nasal obstruction for the past 6 months. Associated symptoms included unilateral, right-sided hyposmia and epistaxis. On physical exam, anterior rhinoscopy revealed a polypoid mass filling the entire right nasal cavity, while the left nasal cavity was unremarkable. Due to the size of the lesion, the point of origin was unable to be determined with in office nasal endoscopy. Computed tomography (CT) of the sinuses without contrast was obtained, revealing an expansile mass within the right nasal cavity that produced downward remodeling of the inferior meatus, near-complete effacement of the middle meatus, leftward septal deviation, and post-obstructive changes of the right maxillary, ethmoid, and frontal sinuses. The anterior skull base and lamina papyracea remained intact and no aggressive osseous destruction was appreciated.

Biopsy of the lesion revealed a diagnosis of sinonasal glomangiopericytoma. The patient was scheduled for definitive surgical excision with right middle turbinate resection, right maxillary antrostomy, right total ethmoidectomy, wide local excision of right nasal septum, and left nasal endoscopy with negative intraoperative margins. The lesion was found to be attached to the posterior-superior septal mucosa. At his most recent clinic visit – 3 years after surgery – he was asymptomatic and without evidence of recurrence.

### Case 2

A 56-year-old male with a history of hypertension presented with a complaint of a known right-sided nasal mass and occasional right nasal pain associated with lifting heavy objects. A year prior to presentation the patient was involved in a motor vehicle accident and imaging incidentally found a right frontal meningioma and a right sinonasal mass. The meningioma was followed by neurosurgery with interval magnetic resonance imaging (MRI), which confirmed the presence of a slow- growing right sinonasal mass. He developed significant right epistaxis which required packing followed by cautery at an outside clinic four months prior to our evaluation. On initial presentation, physical examination was unremarkable. Nasal endoscopy revealed a well demarcated polypoid mass arising from the right sphenoethmoidal recess protruding downward into the nasal cavity to the anterior aspect of the choana. A CT of the sinuses was obtained to better assess skull base anatomy which demonstrated a 3 cm lobulated mass in the posterior right nasal passage as well as a smaller 1 cm mass along the left aspect of the nasal septum. No bony involvement or destruction was noted on imaging.

The patient was scheduled for definitive surgical excision of the mass with right middle turbinate resection, right maxillary antrostomy, right total ethmoidectomy, right superior turbinate partial resection, right sphenoid antrostomy and posterior septectomy with gross total resection of a right posterior septal mass. Intraoperative tumor margins were negative. Final pathology was consistent with a sinonasal GPC on the right and a fibrotic sinonasal polyp on the left posterior septum. To date, three months after complete excision, the patient is without evidence of recurrence.

### Case 3

A 40-year-old male with a history of gastroesophageal reflux disease, obstructive sleep apnea and obesity was referred for evaluation of globus sensation and significant cough for the past 3 months. Flexible nasopharyngolaryngoscopy revealed a bright red polypoid mass attached to the right septum as well as multiple polyps medial to the middle turbinate. Due to the unusual location of the polyp, a CT was obtained which showed partial opacification of a downward recess extending from the right frontal sinus medially along the nasal bone and of the right nasal cavity deep to the middle turbinate as well as bilateral middle concha bullosa without any signs of reactive or destructive bony changes.

He underwent definitive treatment with surgical excision of the right nasal polyp as well as bilateral concha bullosa resection and inferior turbinate reduction. The excised right nasal septal mass was identified as a sinonasal GPC. To date, one month after complete excision, the patient has no evidence of recurrence.

## Discussion

**Figure 1:**
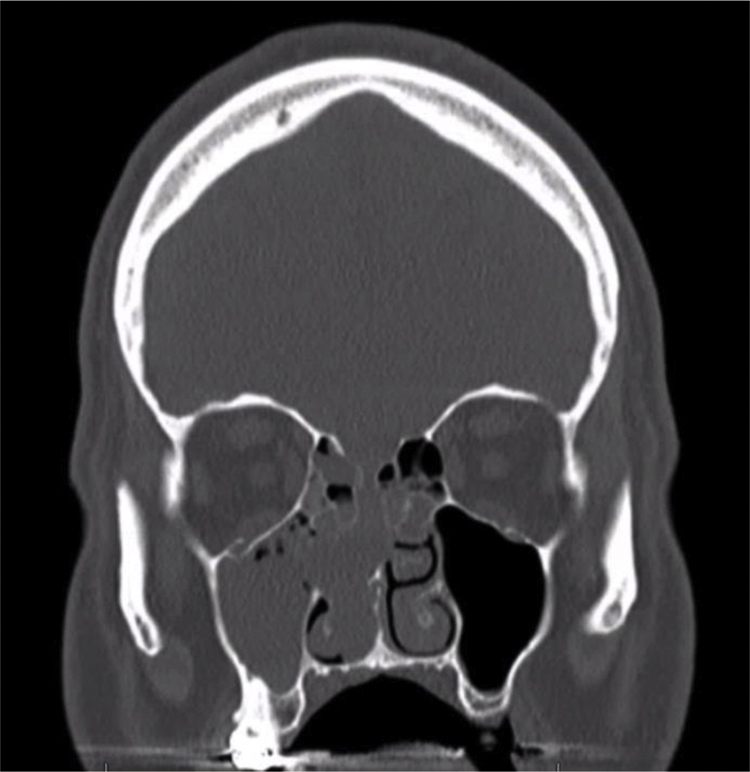
Noncontrast CT. Coronal cut demonstrating complete opacification of the right maxillary sinus with partial opacification of the ethmoid air cells as well as thinning of the skull base.

Sinonasal GPC is a rare entity distinct from other hemangiopericytomas. Grossly, these lesions appear as beefy red or fleshy pink, polypoid, hemorrhagic masses that bleed easily upon palpation.^2^, ^6^ The mean size is approximately 3 cm, but can be as large as 8 cm.^2^ Clinical diagnosis can be challenging, often requiring the use of CT or MRI although the radiographic findings can be nonspecific. On CT these lesions appear as enhancing soft tissue masses, similar in appearance to an inflammatory polyp (Fig. 1). On T1-weighted MRI, the tumor appears as isointense, while it may vary from iso- to hypointense on T2-weighted images.^2^, ^5^

**Figure 2:**
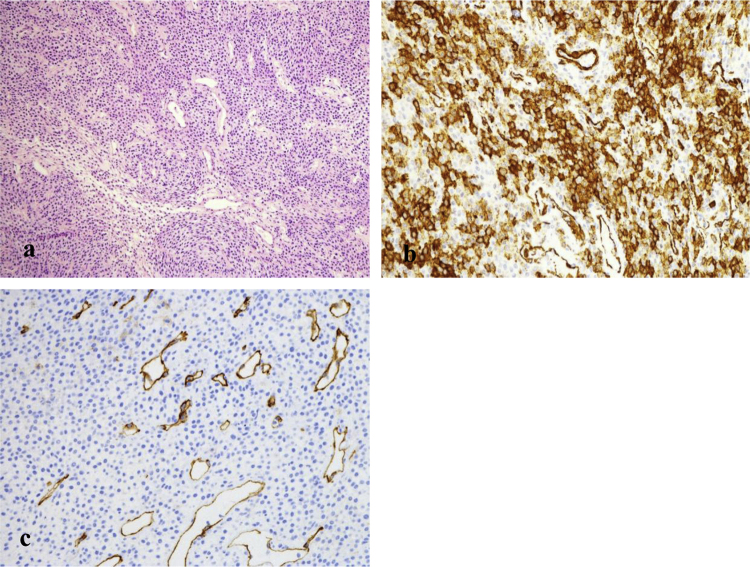
(a) Tightly packed tumor cells with solid and whorled areas asd well scattered staghorn-shaped capillaries. (b) Diffused cellular and cytoplasmic staining for beta-catenin. (c) Staining for CD34 highlights vessels but is negative for tumor cells.

The differential diagnosis for hemorrhagic lesions of the nasal cavity is extensive and includes glomus tumors, angioleoimyoma, lobular capillary hemangioma, solitary fibrous tumor, angiofibroma, and myopericytoma with the final diagnosis requiring histology.^7^, ^8^ Histologically GPC is an unencapsulated, well delineated subepithelial tumor, composed of tightly packed spindle cells growing in fascicles, storiform, whorled, or palisaded patterns. On immunohistochemistry, these cells stain positive for actin, vimentin, and beta catenin but negative for the desmin, keratin, and S10 (Fig. 2a–c).^3^, ^8^ Consistent with their prognosis, GPC histologically has limited mitotic activity and less atypia compared to more malignant, aggressive neoplasms.^6^

These neoplasms tend to be radioresistant but have an excellent prognosis when treated surgically.^3^ The most common approach to treatment of sinonasal GPC involves complete excision with an endoscopic approach. Intracranial extension and pterygopalatine fossa involvement are not contraindications for endoscopic surgical excision of tumor.^5^ Additionally, large, or highly vascular tumors may benefit from preoperative selective embolization prior to endoscopic excision.

Postoperative management should include long term surveillance with nasal endoscopy scheduled at regular intervals, as the local recurrence rate of sinonasal GPC can be up to 40%, with most recurrences occurring within 5 years of excision.^9^ Local recurrences have been shown to occur up to 12 years after initial excision. Moreover, most local recurrences occurring within a year are likely due to incomplete excision.^10^ Rates of metastasis are approximately 5–10% and are generally preceded by multiple recurrences.^5^, ^10^ In the case of metastatic disease, a combination of radiotherapy and chemotherapy may be used as adjunctive or palliative therapy.

## Conclusion

In conclusion, GPC is an uncommon neoplasm of the nasal cavity and paranasal sinuses that must be kept in the differential diagnosis for sinonasal masses. General preoperative workup includes endoscopy, CT, and MRI to assess extent, characteristics, and size of tumor for appropriate preoperative planning. Histological analysis is essential to accurate diagnosis. Complete surgical excision is the treatment of choice, with long term scheduled followup.

## Conflicts of interest

The authors declare no conflicts of interest.
